# Distinct Longitudinal Trajectories of Cognitive Change Among Middle-aged and Older Adults in China

**DOI:** 10.1093/geroni/igaf122.661

**Published:** 2025-12-31

**Authors:** Ying Liu, Dayoung Lee, Stefan Schneider, John Strauss

**Affiliations:** University of Southern California, Los Angeles, California, United States; University of Southern California, Los Angeles, California, United States; University of Southern California, Los Angeles, California, United States; University of Southern California, Los Angeles, California, United States

## Abstract

Monitoring and understanding the longitudinal trajectories of cognitive change is central to dementia care and research. Prior studies that examined the cognitive change in Chinese population either assumed a single trajectory, which ignored interpersonal differences, or modeled change along measurement times rather than age. The current study leveraged a longitudinal, nationally representative survey, the China Health and Retirement Longitudinal Study (CHARLS), and identified distinct trajectories of cognitive change using the Bayesian age-based Growth Mixture Modeling (GMM) techniques. In 2011-2018, four waves of cognition data were collected on 21,242 participants aged 40 years and older, with tasks including time orientation, immediate and delayed word recall, serial’s 7s, and drawing interlocking pentagons. The GMM analyses classified individuals into three subgroups, each with a distinct trajectory as age increased from 40: 65.9% had a high initial cognition with a relatively flat but slightly accelerated decline; 26.8% had a middle initial cognition yet a noticeable and steady decline; and 7.3% had a low initial cognition and a noticeable decline. Adjusting for demographics (education, gender, ethnicity, coupledness, urbanicity based on Hukou or household registration, and migration status), multinomial logistic regression analyses revealed that compared to the high-initial-cognition group, the other two groups tended to be women and have lower education and rural Hukou; the associations were particularly strong for the low-initial-cognition group. These findings provided an insightful view of different patterns in cognitive change among the middle-aged and older Chinese. They also illustrated the potential of the GMM techniques in population research of aging.

